# *CXC motif chemokine receptor 4* gene polymorphism and cancer risk

**DOI:** 10.1097/MD.0000000000005317

**Published:** 2016-12-09

**Authors:** Yang Wu, Chun Zhang, Weizhang Xu, Jianzhong Zhang, Yuxiao Zheng, Zipeng Lu, Dongfang Liu, Kuirong Jiang

**Affiliations:** a Pancreas Center; b The Department of Urology, The First Affiliated Hospital of Nanjing Medical University; c Pancreas Institute, Nanjing Medical University; d Department of Thoracic Surgery, Nanjing Medical University affiliated cancer Hospital; e Jiangsu Key Laboratory of Molecular and Translational Cancer Research, Cancer Institute of Jiangsu Province, Nanjing; f Department of Digestive, Songjiang Branch Hospital of Shanghai First People's Hospital, Nanjing Medical University, Shanghai, China.

**Keywords:** cancer, CXCR4, meta-analysis, polymorphism, rs2228014

## Abstract

**Background:**

Previous epidemiological studies have reported the relationship between CXC motif chemokine receptor 4 (*CXCR4*) synonymous polymorphism (rs2228014), and risk of cancer, but the results remained conflicting and controversial. Therefore, this study was devised to evaluate the genetic effects of the rs2228014 polymorphism on cancer risk in a large meta-analysis.

**Methods:**

The computer-based databases (EMBASE, Web of Science, and PubMed) were searched for all relevant studies evaluating rs2228014 and susceptibility to cancer. In the analysis, pooled odds ratios (ORs) with its corresponding 95% confidence intervals (CIs) were calculated in 5 genetic models to assess the genetic risk. Egger regression and Begg funnel plots test were conducted to appraise the publication bias.

**Results:**

Data on rs2228014 polymorphism and overall cancer risk were available for 3684 cancer patients and 5114 healthy controls participating in 11 studies. Overall, a significantly increased risk of cancer was associated with rs2228014 polymorphism in homozygote model (OR = 2.01, 95% CI: 1.22–3.33) and in recessive model (OR = 1.97, 95% CI: 1.23–3.16). When stratified by ethnicity, the results were positive only in Asian populations (heterozygote model: OR = 1.36, 95% CI: 1.13–1.65; homozygote model: OR = 2.43, 95% CI: 1.21–4.91; dominant model: OR = 1.47, 95% CI: 1.13–1.90; recessive model: OR = 2.25, 95% CI: 1.13–4.48; and allele model: OR = 1.48, 95% CI: 1.10–1.99). Besides, in the subgroup analysis by source of control, the result was significant only in population-based control (homozygote model: OR = 2.39, 95% CI: 1.06–5.40; recessive model: pooled OR = 2.24, 95% CI: 1.02–4.96).

**Conclusion:**

In general, our results first indicated that the rs2228014 polymorphism in *CXCR4* gene is correlated with an increased risk of cancer, especially among Asian ethnicity. Large, well-designed epidemiological studies are required to verify the current findings.

## 1 Introduction

CXC motif chemokine receptor 4 (*CXCR4*) is the exclusive receptor for stromal cell-derived factor-1 (*SDF-1*; *CXCL12*) and is expressed on naive T cells, natural killer cells, dendritic cells, and monocytes.^[1–3]^ Most recently, researchers have focused on *CXCR4* as it is the most common chemokine receptor expressed on cancer cells.^[4]^ It has been suggested that *CXCR4* plays an essential role in tumor progression including colorectal, breast, and oral squamous cell carcinoma, as all of them usually metastasize to *CXCL12*-expressing organs.^[5–8]^ Data from in-vitro experiments as well as from murine in-vivo models, investigating the metastatic capability of *CXCR4*, underlined the vital role of *CXCR4* for cancer cell malignancy, as activation of the 7-transmembrane G-coupled receptor *CXCR4* by *CXCL12* induced proliferation, invasion, migration, and angiogenesis of cancer cells.^[9–12]^

The human *CXCR4* gene is located on chromosome 2q2 and a synonymous polymorphism of *CXCR4*, a cytosine to thymine (C > T), is found at codon 138.^[13,14]^ Several molecular epidemiological studies showed that *CXCR4* was highly mutated and had a pro-oncogenic role in cancers, including renal cell carcinoma,^[15]^ nonsmall cell lung cancer,^[16]^ oral cancer,^[17]^ hepatocellular carcinoma,^[18]^ acute myeloid leukemia,^[19]^ and breast cancer.^[20]^ However, other studies indicated that there was no significant association between rs2228014 polymorphism and cancer risk, such as endometrial carcinoma,^[21]^ bladder cancer,^[22]^ chronic lymphocytic leukemia,^[23]^ breast cancer,^[24]^ and prostate cancer.^[25]^ Because of relatively small sample sizes, these studies provided limited evidence and might have been underpowered to detect the overall effects. Therefore, we conducted this meta-analysis to obtain a more precise estimation from all eligible case–control studies.

## 2 Methods

### 2.1 Primary search strategy

We systematically searched PubMed, EMBASE, and Web of Science comprehensively for all publications regarding the association between the rs2228014 polymorphism and cancer risk (up to June 16, 2016), by using the combinations of the following keywords: CXCR4, rs2228014, variants/polymorphism/polymorphisms/genotype/single nucleotide polymorphism (SNP), and cancer. Additional usable data were hand-searched from reference lists of original studies or review articles. Nevertheless, if several studies were performed on the same subjects, only the 1 with latest, and/or largest sample size would be involved. Ethical approval was not necessary, because this was a meta-analysis, including no direct handing of personal data or recruitment of subjects.

### 2.2 Inclusion criteria and exclusion criteria

Studies involved had to satisfy the inclusion criteria: case–control design was utilized; the diagnosis of the patients with cancer should be pathologically confirmed and the controls were verified as free from any cancer; and sufficient data for estimating an odds ratio (OR) with 95% confidence interval (CI) was available. The major exclusion criteria were as follows: no obtainable genotype frequency data; duplicates of previous publication; and studies designed as a case–case or case-only study.

### 2.3 Data extraction

The identified studies were reviewed separately by 3 of the investigators (YW, CZ, and WX) independently and carefully to determine whether an individual study was eligible for the analysis. These data were extracted from studies involved independently, and the disagreement was resolved by a discussion involving a senior investigator (KJ). All the following data were sought from each study and recorded in a standardized form: first author's name, year of publication, ethnicity of each study population, source of controls, sample size, genotyping method, number of cases, and controls, frequencies of rs2228014 in cases and controls, respectively, and results of the Hardy–Weinberg equilibrium (HWE) test.

### 2.4 Statistical analysis

The pooled ORs with 95% CIs were applied to evaluate the strength of association between rs2228014C/T polymorphism and cancer susceptibility. The fixed-effects model (the Mantel–Haenszel method) and the random-effects model (the DerSimonian–Laird method) were separately employed to pool the data.^[26]^ If existence of heterogeneity was detected, the random-effects model would be more appropriate.

Sensitivity analysis was performed to assess the influence of individual studies on the pooled ORs, with the method of calculating the outcomes again by omitting 1 single study each time. After that, subgroup analyses according to ethnicity, source of controls, and sample size were further carried out to identify the potential association for each subgroup. If total quantity of case and control was larger than 700, the sample size would be regarded as large. The reverse was true for small sample size. Begg funnel plots and Egger linear regression method were taken to estimate the publication bias, and a *P* < 0.05 was set as the significance threshold.^[27]^ HWE was checked by the goodness-of-fit chi-square test, and a *P* < 0.05 was considered as a significantly selective bias.^[28]^

Stata software (version 12.0; StataCorp LP, College Station, TX) was employed in the whole statistical analyses. *P* values were all 2-sided and regarded as statistically significant if less than 0.05.

## 3 Results

### 3.1 Studies characteristics

**Figure 1:**
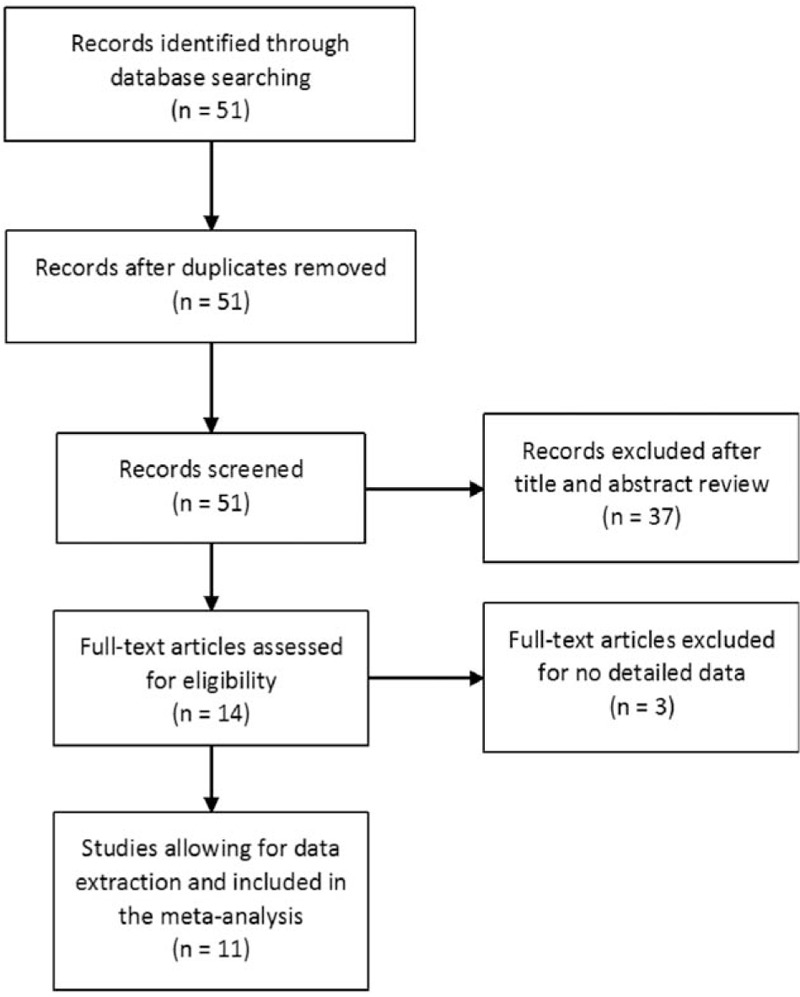
Flow diagram of the study selection process.

The flowchart of study exclusion and inclusion with specific reasons is indicated in Fig. 1. We identified 51 records, among which 14 papers appeared to be potentially eligible for inclusion and were retrieved in full texts. After full-text review, 3 articles were excluded due to no detailed genotyping data (Fig. 1). Therefore, a total of 11 case–control studies including 3684 cases and 5114 controls were ultimately included in the meta-analysis,^[15–25]^ and the details of each study were recorded in Table 1. As a result, each group of them was considered separately for pooling stratified analysis. All studies indicated that the distribution of genotypes in the controls was consistent with HWE except for only 1 study.^[16]^ The sample size ranged between 2831 and 5967. Two genotyping methods were utilized in the studies, including TaqMan and polymerase chain reaction restriction fragment length polymorphism.

**Table 1:**
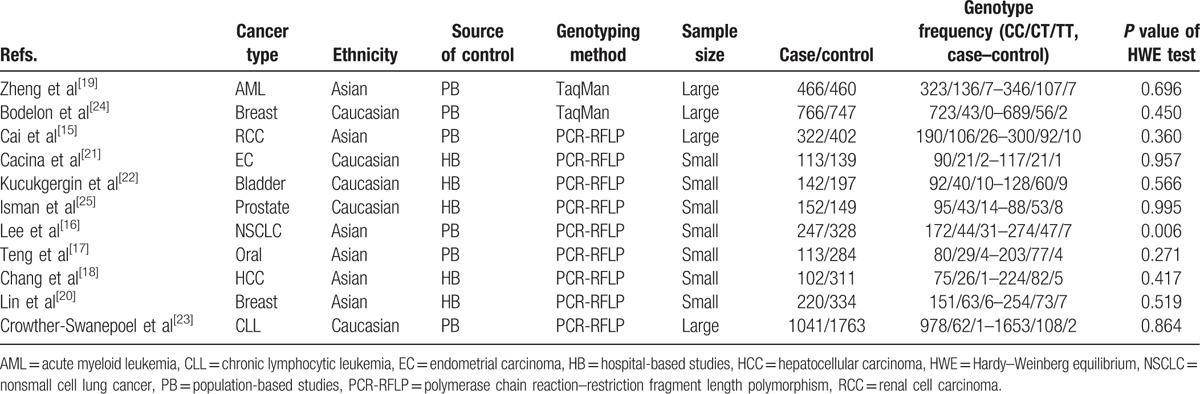
Main characteristics of all studies included in the meta-analysis.

### 3.2 Quantitative synthesis results

**Figure 2:**
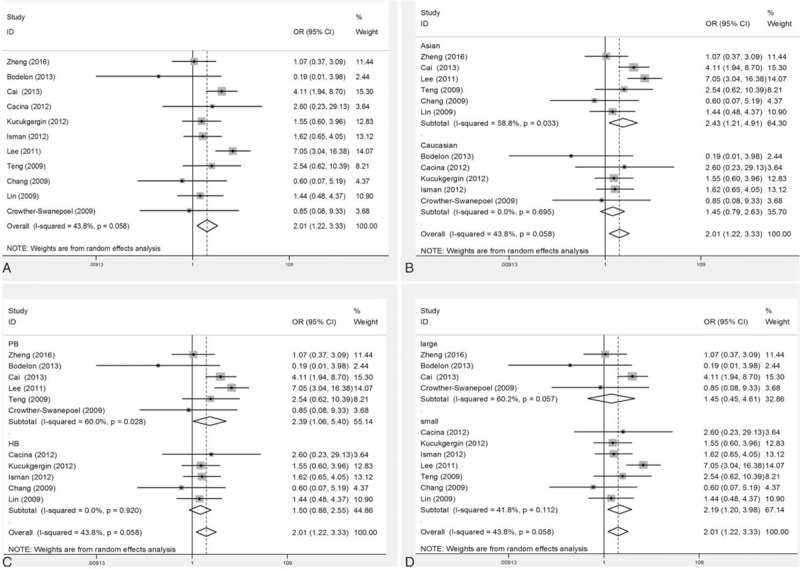
Forest plots of the CXC motif chemokine receptor 4 (rs2228014) polymorphism and cancer risk for overall populations and subgroup analyses under homozygote model (TT vs CC) with random-effects model. ([A] overall cancer risk; [B] ethnicity subgroup; [C] source of control subgroup; and [D] sample size subgroup.).

The key outcomes of the meta-analysis of the association between rs2228014 polymorphism and cancer risk were listed in Table 2. Overall, the pooled OR was 2.01 (95% CI: 1.22–3.33) for homozygote model (Fig. 2A) and 1.97 (95% CI: 1.23–3.16) for recessive model. When the studies were stratified by ethnicity, the results were positive only in Asian populations (heterozygote model: OR = 1.36, 95% CI: 1.13–1.65; homozygote model: OR = 2.43, 95% CI: 1.21–4.91 (Fig. 2B); for dominant model: OR = 1.47, 95% CI: 1.13–1.90; recessive model: OR = 2.25, 95% CI: 1.13–4.48; allele model: OR = 1.48, 95% CI: 1.10–1.99). Moreover, in the subgroup analysis by source of controls, the significant results were detected only in population-based controls (homozygote model: pooled OR = 2.39, 95% CI: 1.06–5.40 (Fig. 2C); recessive model: pooled OR = 2.24, 95% CI: 1.02–4.96). Furthermore, in the stratified analysis by sample size, the significant results were detected in small sample subgroup (homozygote model: pooled OR = 2.19, 95% CI: 1.20–3.98 (Fig. 2D); recessive model: pooled OR = 2.19, 95% CI: 1.24–3.86). As a whole, for *CXCR4* rs2228014 polymorphism association, the carriers of TT genotype held higher cancer risk than carriers of CT/CC genotype, especially in Asian ethnicity.

**Table 2:**
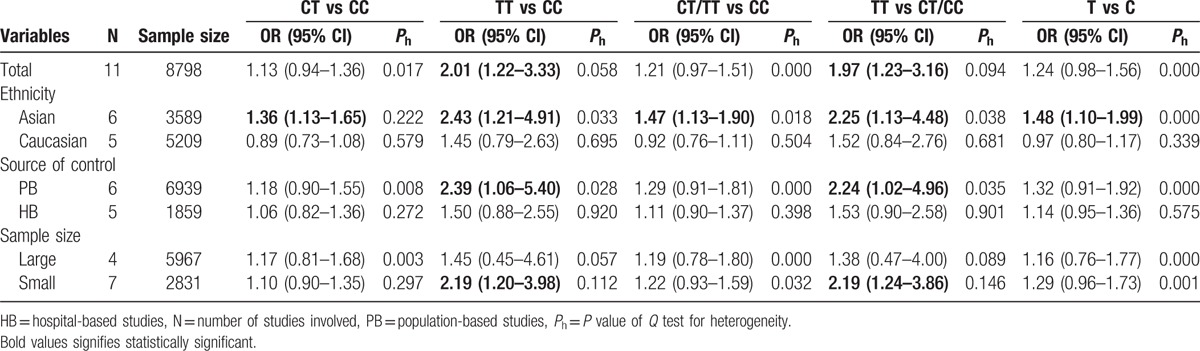
Main results of pooled ORs and stratification analysis of rs2228014 polymorphism on cancer risk in the meta-analysis.

### 3.3 Test of heterogeneity

Heterogeneity between studies was observed in overall genetic models but decreased through subgroup analyses. The analysis of Galbraith manifested that between-study heterogeneity was not prominent.

### 3.4 Sensitivity analysis

**Figure 3:**
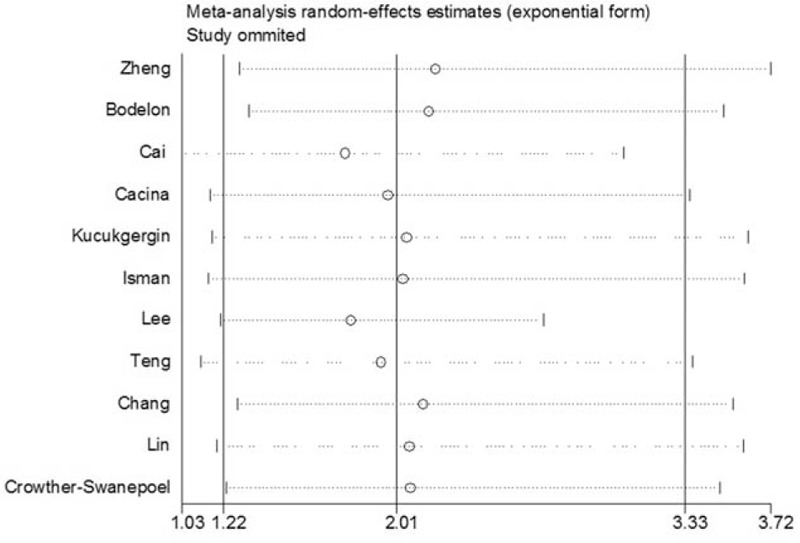
The influence of individual studies on the overall odds ratio under homozygote model (TT vs CC).

We conducted sensitivity analyses by repeating the meta-analysis while sequentially omitting the studies included (1 omitted each time). The sensitivity analysis under homozygote model for rs2228014 polymorphism association in the overall population is shown in Fig. 3, demonstrating that no individual study affected the pooled OR significantly. Although the genotype distribution in 1 study included did not follow HWE,^[16]^ the corresponding pooled ORs were not qualitatively altered when it was omitted. The sensitivity analysis indicated that our results were reliable.

### 3.5 Publication bias

**Figure 4:**
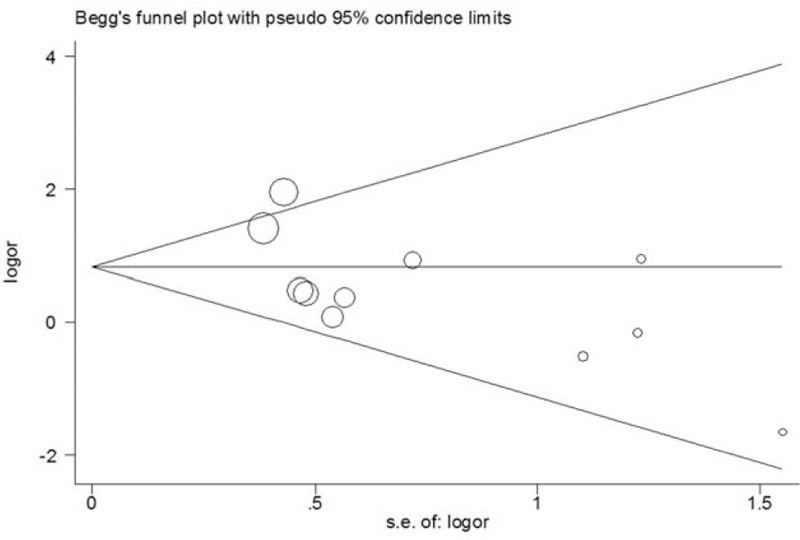
Funnel plots of CXC motif chemokine receptor 4 rs2228014 polymorphism and cancer risk (*P*_Begg_ = 0.161; *P*_Egger_ = 0.062; model: TT vs CC).

To determine the possible publication bias of the literature, the Begg funnel plot was applied and the shapes of them seemed no evidence of obviously asymmetrical, indicating no significant publication bias, which was also further confirmed by Egger test (*P*_Begg_  =  0.161; *P*_Egger_  =  0.062; model: TT vs CC). The overall results revealed that our results were statistically reliable (Fig. 4).

## 4 Discussion

As one of the important causes of population diversity, SNP is the most common type of human genetic variation associated with cancer susceptibility.^[29]^ Recently, much research effort has been directed toward understanding the role of SNPs located in chemokine receptor gene and their influences on susceptibility to and progression of various diseases. Clarifying the association between chemokine receptor gene *SNPs* and cancer risk will benefit to further illuminate the mechanism underlying the carcinogenesis, which will in turn provide novel biomarkers for screening high-risk populations for cancer and promoting the development of molecular-targeted therapy.

As the exclusive receptor for CXCL12, CXCR4 has been verified to play an essential role in the progression and prognosis of tumor.^[9–12,30,31]^ The *CXCR4* gene is located on chromosome 2q2, and a synonymous polymorphism of CXCR4 (I138I) have been identified that may influence cancer risk.^[13,14]^ Although the hypothesis of the neutral theory of molecular evolution—that some categories of mutation, like synonymous mutations, have too small an effect on fitness to be affected by natural selection—seems intuitively reasonable, over the past few decades the theory has been not applicable. New evidence indicates that even some synonymous mutations are subject to constraint, often because they affect splicing and/or messenger RNA stability.^[32]^

To date, many case–control studies that have been carried out to investigate whether *CXCR4* rs2228014 polymorphism is associated with the risk of cancer have yielded conflicting results,^[15–25]^ which might partially own to the relatively small sample size of individual study, the different distributions of patients or controls, different cancer types, and the various methodologies. Meta-analysis as a powerful tool can provide more reliable results than a single study especially in explaining controversial conclusions.^[33]^ To achieve a better understanding of the association between rs2228014 and cancer risk, we conducted this meta-analysis with larger sample size and subgroup analyses, which provided new evidence for the susceptibility and etiology of cancer. To the best of our knowledge, our meta-analysis, on the basis of 11 case–control studies (3684 cancer patients and 5114 healthy controls), is the first, also the largest and most comprehensive assessment to investigate this association and the final results showed that rs2228014 polymorphism was associated with an increased cancer risk.

Interestingly, stratified analysis by ethnicity indicated that, among Asians, the rs2228014 polymorphism was significantly associated with increased cancer risk under all the 5 genetic models. The differences between Asians and other races may be partly due to the different genetic backgrounds and environments or lifestyles. In the subgroup analysis of different sources of controls, evidence of an association between the rs2228014 polymorphism and an increased cancer risk was found in the population-based studies, but not in the hospital-based studies (HB). In the stratified analysis of ethnicity, our results showed that the T allele of rs2228014 had a 2.01-fold risk of cancer in overall populations, a 2.43-fold risk (95% CI: 1.21–4.91) in Asian populations. Lack of association among HB subgroup was probably due to the fact that the controls recruited from hospital could not represent the general population well. In the future, well designed studies with large sample size might provide more precise results about such associations.

Some advantages could be emphasized in our meta-analysis. First, this research shed lights on the relationship of a synonymous polymorphism in *CXCR4* gene and the increased susceptibility to human cancers, especially in Asian population. Second, the comprehensive inclusion criteria and articles on wide range of cancers enhanced the power and persuasion of our conclusion. Lastly, no publication bias was detected, indicating that the results might be unbiased.

Despite the overall sufficient and robust statistical evidence generated through this analysis, some limitations should be addressed. To begin with, in the stratified analyses, the sample size of some subgroups was relatively small, with limited statistical power to explore the real association. In addition, included studies are still so limited that we cannot perform subgroup analysis for different cancer types although it is well known that 1 SNP may act different roles in different kinds of cancer. What is more, the lack of original data in some valuable researches restricted us to continue investigating some potential interactions, such as age, sex, family history, environmental factors, and lifestyle. Accordingly, it is required that a more precise analysis could be performed if individual data were available.

## 5 Conclusion

The outcomes of the present meta-analysis demonstrate that the *CXCR4* gene rs2228014 polymorphism might be a potential detecting index for the risk of cancer in the future. In order to acquire a more integrate understanding of the correlation between rs2228014 polymorphism and cancer susceptibility, several recommendations have been suggested as follows: (first) though the sufficient evidence has been achieved in this meta-analysis, more studies by standardized unbiased methods are required, which can provide more detailed individual data of high quality. (second) Effects of different gene polymorphisms need to be combined, due to the intricate genetic background of cancer development, including single-candidate genes as well as complex epigenetic process. In consequence, a single gene polymorphism is difficult to be identified responsible for cancer. (third) More comprehensive and generalizable conclusions are expected to achieve among various ethnic groups.

## Acknowledgments

The authors thank Dr Xiao Li of the Department of Urology, The First Affiliated Hospital of Nanjing Medical University, for his contributions and guidance.
